# Methodological concerns on the association between frailty and chronic lung disease

**DOI:** 10.1007/s40520-025-02930-y

**Published:** 2025-05-21

**Authors:** Hong Fan, Wenwen Zhao, Ying Chen, Yan Yang

**Affiliations:** Pulmonary and Critical Care Medicine, Shandong Public Health Clinical Center, Shandong, China

**Dear Editor:**

We read with interest the study by Feng et al. examining the association between frailty status and chronic lung disease (CLD) risk using China Health and Retirement Longitudinal Study and English Longitudinal Study of Ageing cohorts [1]. While this study provides valuable insights, several important methodological issues warrant attention.

First, we noticed an intriguing yet unexplained finding that younger frail individuals (< 65 years) showed a higher risk of CLD (HR = 1.87) compared to older adults. This observation appears paradoxical given that frailty is traditionally conceptualized as a geriatric syndrome [2]. The authors should have explored potential mechanisms underlying this association, such as whether early-onset frailty represents a distinct phenotype with different pathophysiological pathways, or whether this finding reflects detection bias or healthcare-seeking behaviors among younger adults. This unexpected age-related pattern deserves thorough discussion rather than being overlooked.

Second, the study’s covariate selection raises concerns about potential unmeasured confounding. The analysis primarily adjusted for sociodemographic factors while omitting crucial lifestyle and clinical variables. Notably absent is physical activity, which is intrinsically linked to both frailty transitions and respiratory health [3]. Furthermore, the lack of adjustment for comorbidities such as hypertension and diabetes, which are established risk factors for CLD [4], represents a significant limitation. This incomplete adjustment strategy might explain the unexpected lack of association between frailty and CLD risk among older adults in the CHARLS cohort.

Third, the authors’ approach of categorizing the frailty index into three groups (robust, pre-frail, and frail) may have obscured the true nature of the association between frailty index and CLD risk. We suggest that restricted cubic spline analysis would have been more appropriate to explore potential non-linear relationships [5]. This method could reveal threshold effects or complex patterns in the association that are masked by simple categorization, providing more nuanced insights for clinical practice.

These methodological refinements would strengthen the evidence base for using frailty assessment in CLD risk prediction and intervention planning.

## Data Availability

No datasets were generated or analysed during the current study.
